# Implementation and success factors from Thailand’s 1-3-7 surveillance strategy for malaria elimination

**DOI:** 10.1186/s12936-021-03740-z

**Published:** 2021-04-27

**Authors:** Cheewanan Lertpiriyasuwat, Prayuth Sudathip, Suravadee Kitchakarn, Darin Areechokchai, Sathapana Naowarat, Jui A. Shah, David Sintasath, Niparueradee Pinyajeerapat, Felicity Young, Krongthong Thimasarn, Deyer Gopinath, Preecha Prempree

**Affiliations:** 1 Division of Vector Borne Diseases, Department of Disease Control, Ministry of Public Health, Nonthaburi, Thailand; 2 Inform Asia: USAID’s Health Research Program, RTI International, Bangkok, Thailand; 3 U.S. President’s Malaria Initiative, United States Agency for International Development (USAID), Regional Development Mission for Asia, Bangkok, Thailand; 4 Independent Consultant, Nonthaburi, Thailand; 5 World Health Organization, Nonthaburi, Thailand; 6 Department of Disease Control, Ministry of Public Health, Nonthaburi, Thailand

**Keywords:** Elimination, Surveillance, 1-3-7 strategy

## Abstract

Thailand’s National Malaria Elimination Strategy 2017–2026 introduced the 1-3-7 strategy as a robust surveillance and response approach for elimination that would prioritize timely, evidence-based action. Under this strategy, cases are reported within 1 day, cases are investigated within 3 days, and foci are investigated and responded to within 7 days, building on Thailand’s long history of conducting case investigation since the 1980s. However, the hallmark of the 1-3-7 strategy is timeliness, with strict deadlines for reporting and response to accelerate elimination. This paper outlines Thailand’s experience adapting and implementing the 1-3-7 strategy, including success factors such as a cross-sectoral Steering Committee, participation in a collaborative regional partnership, and flexible local budgets. The programme continues to evolve to ensure prompt and high-quality case management, capacity maintenance, and adequate supply of lifesaving commodities based on surveillance data. Results from implementation suggest the 1-3-7 strategy has contributed to Thailand’s decline in malaria burden; this experience may be useful for other countries aiming to eliminate malaria.

## Background

In line with the World Health Organization (WHO) Global Technical Strategy for Malaria 2016–2030 (GTS) and the subsequent Framework for Malaria Elimination and Malaria Surveillance, Monitoring and Evaluation reference manual [1], the countries in the Greater Mekong Subregion (GMS) have endorsed the goals of eliminating *Plasmodium falciparum* by 2025 and all forms of malaria by 2030 [1]. The GMS comprises Cambodia, Lao People’s Democratic Republic, Myanmar, Vietnam, and Thailand, which are all showing notable declines in malaria burden [2]. GMS countries are approaching the challenge using several tailored malaria elimination strategies, including distribution of insecticide-treated bed nets (ITNs), expanded networks of village health workers, and case-based surveillance and response [3, 4].

Thailand’s National Malaria Elimination Strategy 2017–2026 (NMES), which outlines a vision for reaching zero indigenous cases by 2024, was approved by the Cabinet of the Royal Thai government in 2016. A complementary Operational Plan 2017–2021 proposed a national budget of 2.3 billion Thai baht (USD $64.8 million) over the next 5 years to support malaria elimination strategies to interrupt transmission [5]. The core focus of these plans is to identify infections rapidly and to use timely and active surveillance and response to prevent them from spreading [5]. The NMES introduced the 1-3-7 strategy as a robust surveillance and response approach for elimination that would prioritize timely, evidence-based action. The strategy requires notification of each malaria case within 1 day of diagnosis, investigation and classification of each case within 3 days, and a focus investigation and response within 7 days to deploy needed interventions.

The 1-3-7 strategy builds on Thailand’s history since the 1980s of conducting case investigation and classification. The Containment Project, which was implemented from 2008 to 2012, served as precursor to real-time case-based data collection and follow-up and led to the launch of electronic data capture in a national web-based malaria information system (MIS) known as “Malaria Online [6]”. In 2009, Thailand added foci investigation as a central part of its malaria control strategy [7]. With the launch of the NMES in 2017, Thailand’s Division of Vector Borne Diseases (DVBD) within the Department of Disease Control (DDC) of the Ministry of Public Health (MOPH) upgraded Malaria Online to aggregate all sources of malaria case data and monitor progress on a near real-time basis [8].

The 1-3-7 malaria elimination strategy was initially developed and implemented in China in 2012 [9, 10], and Thailand both studied and adapted the strategy for the local epidemiological context. The strategy is considered a key factor in helping China reach zero locally transmitted malaria cases in 2017 and in supporting its efforts to maintain the interruption of transmission. Other countries in Southern Africa and Southeast Asia have also established malaria case reporting and response follow-up systems, complemented by increased monitoring activities [11, 12]. However, the hallmark of the 1-3-7 strategy is timeliness, with strict deadlines for reporting and response to enforce a robust elimination programme. This paper outlines Thailand’s experience adapting and implementing the 1-3-7 strategy, including lessons learned and success factors, in the hope that this information may be helpful for other countries considering strengthening surveillance for malaria elimination.

### From malaria control to malaria elimination

From 2012 to 2015, the DVBD’s malaria control efforts were associated with a significantly reduced blood slide positivity rate (SPR) of less than 5% among suspected cases with fever and an annual parasite incidence (API) of < 1 per 1000 population (range 0.38–0.82) [5]. The same downward trend was seen in malaria incidence, decreasing from 33,835 cases in 2012 to 24,332 cases in 2015, representing a 28.1% decline in just 3 years [8]. The malaria control programme also documented reduced mortality, with just 0.05 deaths per 100,000 population by 2015, representing fewer than 50 malaria-attributed deaths from 2012 to 2015.

**Fig. 1:**
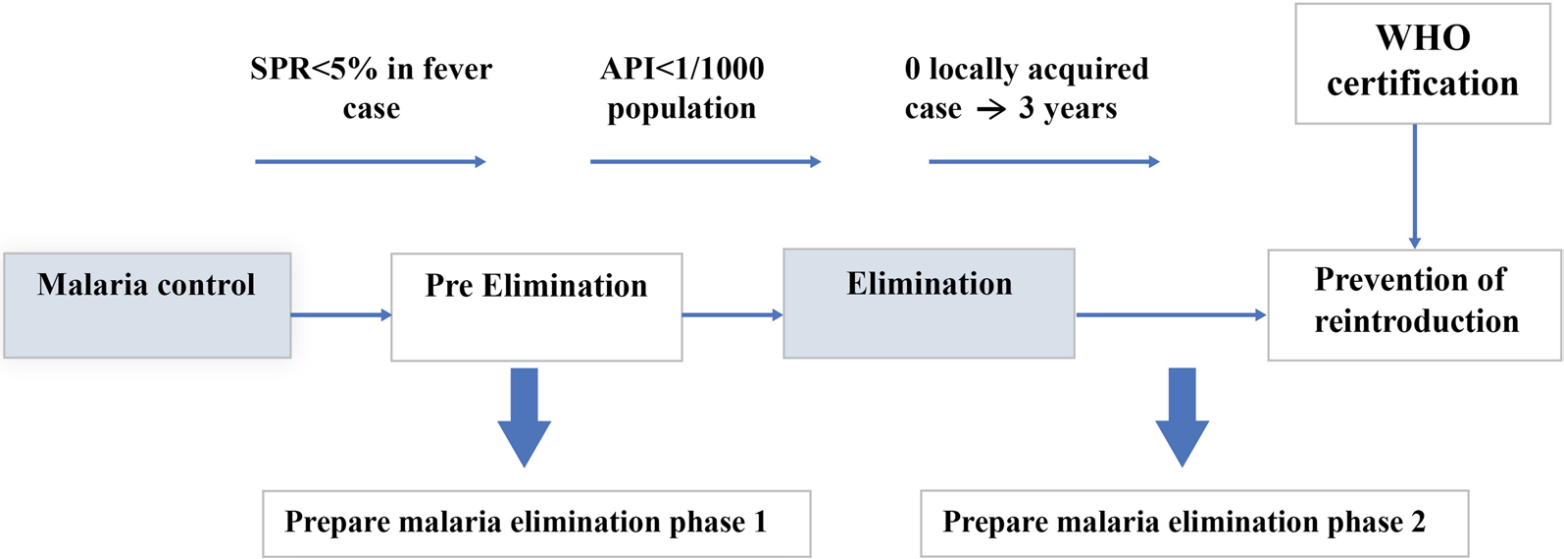
Milestones from malaria control to malaria elimination. (Asterisk) Source: Malaria Elimination Strategic Plan for Thailand 2017–2026 [5]. *API* annual parasite incident, *SPR* slide positive rate

These results led to a comprehensive malaria programme review in 2015 to identify strengths, gaps, and challenges across disciplines and to recommend key actions [5, 13]. Recommendations for surveillance included updating and reorienting surveillance as a core intervention to detect and trigger an appropriate, focused, and timely response for every confirmed malaria case. Also in 2015, malaria became a notifiable disease in Thailand with the passage of the Infectious Disease Act, which expanded surveillance potential. To improve performance, the DDC decided to transition the malaria programme to one focused on elimination (Fig. 1) per the Global Technical Strategy for Malaria 2016–2030, with a goal to achieve national malaria elimination by 2024 [1]. The MOPH created a Malaria Elimination Strategic Plan for Thailand 2017–2026, and the DDC contributed the complementary Thailand Malaria Elimination Operational Plan 2017–2021 to pave the way forward [5]. These plans outlined incremental targets to support zero malaria by 2024.

The DVBD developed a new district-level stratification of malaria endemicity based on guidance from the WHO Malaria Elimination framework [14], which is detailed in the NMES [5]. The stratification is the basis for subsequent tailored case and vector management activities for elimination based on evidence from surveillance data. The DVBD also coordinated adoption of existing systems and infrastructure to prepare for adoption of the 1-3-7 strategy, including strengthening and sustaining epidemiological and entomological surveillance as recommended by the malaria programme review committee.

### Protocols of the 1-3-7 strategy

#### Case reporting within 24 h

All individuals who test positive for malaria using microscopy or a rapid diagnosis test (RDT) receive malaria treatment according to the national treatment guidelines and trigger the start of the 1-3-7 surveillance process. All malaria species are included in 1-3-7 surveillance efforts, with *P. falciparum* and *Plasmodium vivax* being most common in Thailand [8]. Health care providers were already required to report confirmed cases and laboratory results under Thailand’s malaria control strategy [8]; this was adjusted for the elimination programme to include a requirement to complete the notification within 24 h (1 day) of diagnosis [15]. An optional cellphone-based short message service alert system immediately informs district-level staff of a case, ensuring timely follow-up. Providers may also choose to report cases using the traditional paper form.

#### Case investigation and classification within 3 days

Thailand has long conducted malaria case investigations to identify risk behaviour and to classify cases as imported or indigenous. Under the NMES, the MOPH adopted an enhanced case classification to allow a more precise understanding of where the patient contracted the disease and identify a source of transmission from remaining hotspots. Thailand’s case investigation form (EP3) includes minimal essential data (e.g., demographics, illness history, diagnostic results, treatment, travel history) and likely location of infection. Cases are investigated by malaria clinic workers, district officers, or provincial officers and are classified per the criteria in Table 1 [4].

**Table 1:** Case classification in Thailand

Case classification	Current definition
A	Indigenous case, a patient who contracted malaria in the village where the patient lived during infection period
B	Imported case, a patient who contracted malaria from another area. Place of infection must be identified at village level
Bx	Contracted malaria from another village but within the same subdistrict
By	Contracted malaria from another subdistrict but within the same district
Bz	Contracted malaria from another district but within the same province
Bo	Contracted malaria from another province
Bf	Contracted malaria from abroad
C	Relapse case, a *Plasmodium vivax* or *Plasmodium ovale* patient who had a malaria episode and has recurrent fever that is not a new infection
D	Induced case, a patient who received malaria parasites from blood transfusion
E	Introduced case, a patient who contracted malaria with evidence connecting the case to an imported case
F	Unclassified case where, after investigation, staff are unable to determine a whether the patient is A or B
G	Uninvestigated case, a patient that staff unsuccessfully attempted to investigate

Case investigation data are recorded on a paper form that is then uploaded to a national repository in the MIS. If the conclusive case classification is “A,” a focus investigation is warranted, and the surveillance rapid response teams (SRRT) deploys the focus investigation and response.

#### Foci investigation and response within 7 days

Thailand’s foci investigation compiles epidemiological, entomological, and sociological information and the results of past interventions. This allows local health authorities to identify causes and characteristics of indigenous transmission to deploy appropriate interventions. With the NMES, Thailand adopted a new foci stratification with four categories according to level of transmission, as outlined in Table 2 [16]. Thailand’s adoption of the NMES also reoriented foci investigation in two ways. Firstly, a strict timeline to conduct the investigation within 7 days of case investigation was required. Secondly, in active foci (A1 and A2 areas) where all interventions are applied, but malaria cases persist for over 4 consecutive weeks, reactive case detection (RACD) is conducted (Table 3). RACD is a surveillance response initiated when an indigenous index case is found in a village (foci) with transmission (A1 or A2) or without transmission but with the presence of a suitable vector (B1). Blood is taken for microscopy from all members living in the index patient’s house and all neighbors living around the index patient’s house, aiming for at least 50 blood samples or 10 households within a 1-km radius. Additional foci response activities include vector control (distributing ITNs to reach 90% coverage at 1 bed net per 2 persons or indoor residual spraying [IRS] of at least 90% of households) and health education [4]. These data are stored in the MIS in a designated foci registry.

**Table 2:** Foci classification in Thailand

Foci classification	Current definition
A1	Active foci: village with indigenous cases in the current year
A2	Residual non-active foci: village without indigenous cases in the current year but with indigenous cases in the previous 3 years
B1	Cleared foci but receptive: village without indigenous cases for 3 consecutive years but vectors are found or environment is suitable for vector breeding
B2	Cleared foci but not receptive: village without indigenous cases for 3 consecutive but vectors are not found or environment is not suitable for vector breeding

**Table 3:** Implementation of the 1-3-7 strategy for malaria elimination

1-3-7 strategy		Activities					
Case notification (within 1 day)		Confirmed malaria cases					
Area classification		Active foci (A1)	Residual non-active foci (A2)	Receptive foci (B1)		Non-receptive foci (B2)	
Case investigation and classification (within 3 days)		Indigenous/Imported	Indigenous/Imported	Imported	Indigenous		Imported
Foci investigation and response (7 days)	Surveillance	RACD 50 people/10 HHs 1–2 km PACD (2 rounds/year) Foci investigation (persistent indigenous cases)	RACD 50 people/10 HHs 1–2 km PACD (1 round/year) Foci investigation (if an indigenous index case is reported)	RACD 50 people/10 HHs 1–2 km Re-investigation and confirmation	RACD in whole village and cluster screening Re-investigation and confirmation Foci investigation Confirm new focus		Continue surveillance
	Diagnosis	Community^a^ (RDT/Microscopy) Public health facility^b^ (Microscopy)	Community (RDT/Microscopy) Public health facility (Microscopy)	Public health facility (Microscopy)	Community (RDT/Microscopy) Public health facility (Microscopy)		Hospitals (Microscopy)
	Treatment and follow-up	Supervised treatment Case follow-up^c^	Supervised treatment Case follow-up	Supervised treatment Case follow-up	Supervised treatment Case follow-up		Supervised treatment Case follow-up
	Vector control	Vector survey ITNs or IRS (90% coverage) LLIN (1 per 2 persons)	Vector survey ITNs or IRS (90% coverage) LLIN (1 per 2 persons)	Vector survey	Vector survey ITNs or IRS (90% coverage) LLIN (1 per 2 persons)		

*HH* household, *IRS* indoor residual spraying, *ITN* insecticide-treated net, *LLIN* long-lasting insecticidal net (includes ITNs, retreated bed nets, and hammocks), *PACD* proactive case detection, *RACD* reactive case detection, *RDT* rapid diagnosis test

^a^Malaria posts and malaria clinics

^b^Health promoting hospitals and district, provincial, and regional public hospitals

^c^Follow-up and re-testing for *P. falciparum* and *P. vivax* cases according to national guidelines

### Enhancing surveillance infrastructure for elimination

**Fig. 2:**
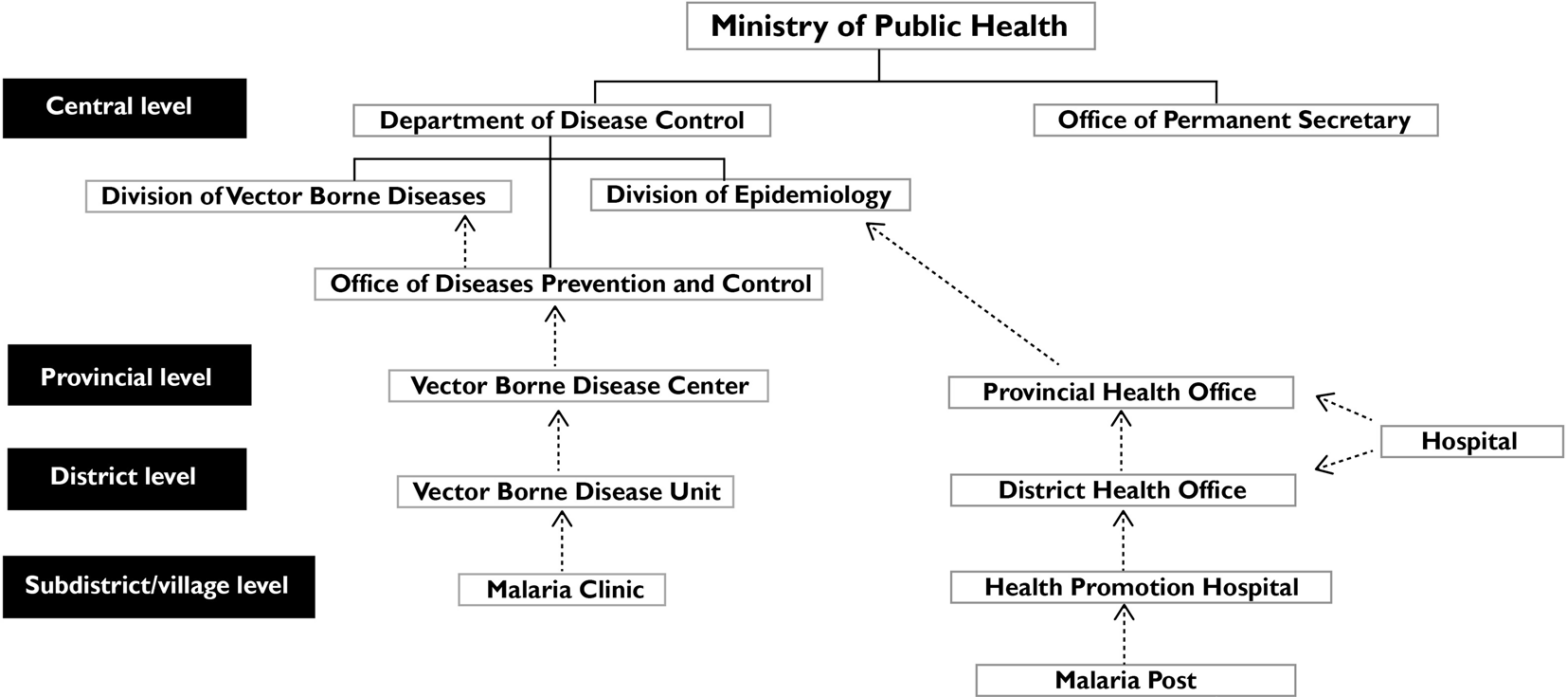
Flowchart of malaria reporting. Source: DVBD [8], MOPH [17]

Malaria Online was introduced in 2012 to facilitate case-based surveillance data tracking through online and offline reporting. The MIS comprises real-time malaria case data, geospatial foci and vector mapping, and drug efficacy data. It also serves as a resource portal for policies and strategic documents related to malaria. The MIS collates two main sources of malaria case recording and reporting (Fig. 2):

1. The vertical malaria programme, starting from subdistrict malaria clinics (MCs) through provincial Vector Borne Disease Centres (VBDCs). These health facilities record data on paper forms for case notification and case investigation; district health teams then input the data into the MIS.
2. The general health services (GHS) programme, starting from community-level malaria posts (MPs) and hospitals (all types) through Provincial Health Offices (PHOs) to the Bureau of Epidemiology (BOE) [8]. These health facilities record data in a separate system maintained by the BOE.

Thailand is currently in a semi-vertical approach for malaria elimination as these two reporting lines are being integrated. Regardless of data type, 1-3-7 guidelines for timeliness of reporting are enforced.

**Fig. 3:**
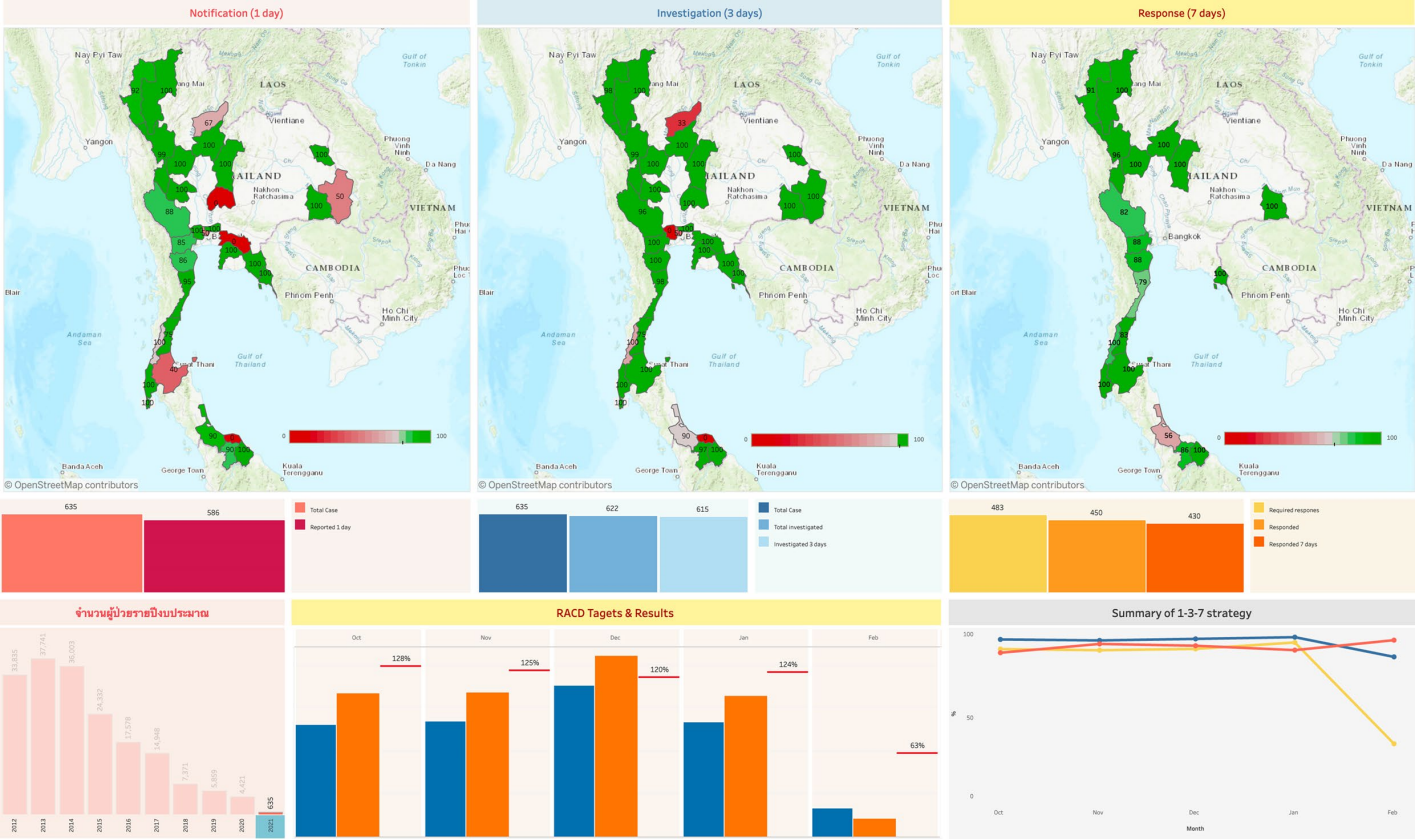
1-3-7 dashboard in the MIS. Source: MIS database [8], DVBD, MOPH

In 2016, new features were added to enable real-time monitoring at every level of the health system and to encourage local staff to take ownership of their data and use the results to improve intervention targeting. The DVBD installed dashboards and visualizations in the MIS to show geospatial mapping of the 1-3-7 metrics, RACD targets and achievements, and a summary of the 1-3-7 malaria elimination strategy (Fig. 3). A new 1-3-7 dataset, representing information from the weekly and monthly reports generated by provinces, includes the number of malaria cases reported and the proportion reported within 1 day, the number of cases investigated and the proportion investigated within 3 days, and the number of foci investigations (with RACD) and the proportion investigated within 7 days. From the first year of implementation through fiscal year 2020, on-time case notification improved from 24.4% to 87.8%, case investigations from 58.0% to 94.7%, and foci investigations from 37.9% to 84.1%. Detailed results on these key performance indicators will be reported separately. To encourage local staff to adopt 1-3-7 protocols and use the resulting data, the DVBD also created a weekly report that collates data highlights.

**Fig. 4:**
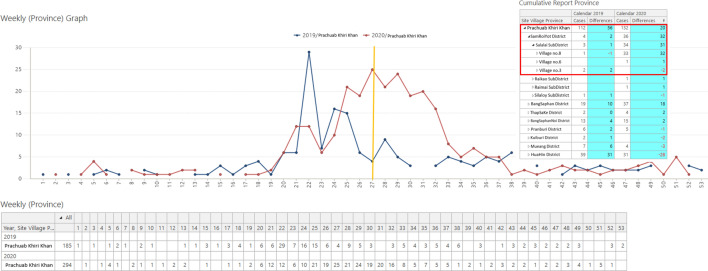
Weekly data report for Prachuab Khiri Khan in the MIS. Source: MIS database [8], DVBD, MOPH

By allowing for timely analysis of malaria surveillance and case investigation data, Malaria Online supports Thailand’s goal to use surveillance to develop strategic information that leads to appropriate action. A Business Intelligence add-on to the MIS facilitates comparisons of weekly incidence across years. If case numbers are higher than expected, the DVBD or provincial authorities alert the relevant subdistricts to consider appropriate investigation and control activities. In the example below, Prachuab Khiri Khan Province received notice during the 27th week of the year that there were 20 more cases reported than in the same week the previous year (Fig. 4). The notice included a dropdown list of villages; the action required to find out which areas were affected; and instructions for prompt case re-investigation, focus investigation with RACD of the whole village, and renewed vector control measures in line with the 1-3-7 strategy. Subnational officers took prompt action during the 27th week, with new cases triggering additional case investigations and a wider response until the next week witnessed a decreasing number of malaria cases.

For data to inform more granular operational decision-making as malaria burden decreases, data must be timely and accessible to subnational officers, health workers, and community focal persons. However, in the remaining hotspot locations in Thailand internet access may be unreliable and, therefore, prevents access to submitting and obtaining data in real time. To address these disadvantages, at the provincial and district levels the MIS can access and store back-up data in the Malaria Offline database in a central server [8]. Malaria Offline complements Malaria Online to ensure that work can continue during internet outages and that key staff can access their data.

### Human resources for strong surveillance

Malaria surveillance for elimination in Thailand relies on human resources in both the malaria vertical programme and the GHS system (Table 4). The surveillance system also engages village health volunteers (VHVs) for community reporting on 1-3-7 metrics. Some Vector Borne Disease Units (VBDUs) supervise GHS staff to ensure high-quality malaria case detection, surveillance, and response.

**Table 4:** Roles and responsibilities in implementing the 1-3-7 strategy

Country level	Ministry of Public Health	
	Division of Vector Borne Diseases, Department of Disease Control	Department of Epidemiology
Central level	Make policy decisions Supervise, monitor, and evaluate interventions Integrate data from all sources to the MIS database Cross-check and analyse data; share feedback Share feedback with and support subnational staff Provide technical support for the MIS and Malaria Offline Collaborate with Department of Epidemiology as C-SRRT	Make policy decisions Cross-check and analyse data Share feedback with and support subnational staff Collaborate with Department of Disease Control as C-SRRT
Provincial level	Vector borne disease centers Enter and upload data (Days 1, 3, 7) into Malaria Offline program Supervise, monitor, and evaluate interventions Monitor provincial MIS data and facilitate reporting and feedback Collaborate with Provincial Health Office as P-SRRT	Provincial health offices Supervise, monitor, and evaluate interventions Monitor provincial data and facilitate reporting and feedback Collaborate with Vector Borne Disease Center as P-SRRT
District level	Vector Borne Disease Units Enter and upload data (Days 1, 3, 7) into Malaria Offline program Supervise, monitor, and evaluate interventions Monitor district MIS data and facilitate reporting and feedback Collaborate with District Health Office as D-SRRT Day 1: send malaria case reports (EP1) to provincial and central levels Day 3: complete case investigation and classification; send EP3 form to district level	District health offices Enter data into HosXP reporting program Supervise, monitor, and evaluate interventions Monitor district data and facilitate reporting and feedback Collaborate with Vector Borne Disease Units as D-SRRT
		District hospitals Conduct malaria testing, treatment, and follow-up Day 1: send malaria reports (R506) to District Health Office Day 3: complete case investigation and classification; send form to Department of Epidemiology Collaborate with Vector Borne Disease Units as D-SRRT
Subdistrict level	Malaria clinics Conduct malaria screening, testing, treatment, and follow-up Day 1: send malaria case reports (EP1) to district level Day 3: complete case investigation and classification; send EP3 form to district level Day 7: conduct foci investigation and RACD as L-SRRT	Health promoting hospital, health centers Supervise, monitor, and evaluate interventions Conduct malaria testing, treatment, and follow-up Day 1: send malaria reports (R506) to district level Day 3: complete case investigation and classification; send form to Department of Epidemiology Day 7: conduct foci investigation and RACD as L-SRRT
Community or village level	Malaria posts and border malaria posts Conduct malaria screening, testing, and follow-up Day 1: send malaria case report (EP1) to district level Day 7: support L-SRRT	Village health volunteers Conduct malaria screening, testing, and follow-up Day 1: send malaria case report (EP1) to district level Day 7: support L-SRRT

EP1, Malaria Blood Examination form; EP3, Investigation and Radical Treatment of Malaria case form; R506, Disease Surveillance Report form

*D-SRRT* district-SRRT, *L-SRRT* local-SRRT, *MIS* malaria information system, *SRRT* surveillance rapid response team, *P-SRRT* provincial-SRRT

Day 1 malaria cases could be found and notified by staff from MCs, MPs, or border posts or by general health facility clinicians. Day 3 case investigations are conducted by MC staff or sometimes by public health workers at hospitals or MP staff with additional follow-up from SRRTs. Day 7 foci investigation and response largely relies on district-level SRRTs, a national system of epidemiology and investigation teams established in 2004 [18]. SRRT members include staff of both the vertical and GHS programmes.

Both the DVBD and BOE remain committed to building skills and updating training materials and guidelines for effective implementation of the elimination programme and the 1-3-7 strategy. The training package targeted epidemiologists, malaria surveillance focal points, and entomologists and was complemented by annual refresher training [13]. Public health workers trained all epidemiologists as part of the SRRT launch in each province to integrate malaria elimination activities into their work, including the 1-3-7 strategy and other public health responses. The training, comprising both workshops and on-the-job coaching, covered MIS data monitoring for timely action to correctly identify malaria infections and disrupt transmission. Since the launch of the 1-3-7 strategy, the DVBD has conducted annual trainings, resulting in 719 trained epidemiologists in Thailand. At the subdistrict and community levels, training for healthcare providers included VHVs. Entomologists were trained on vectors present in each subdistrict or VBDU area, a collection of mosquitoes for polymerase chain reaction (PCR) testing to rule out indigenous transmission in new B1 areas prior to their designation, and insecticide resistance monitoring for endemic areas.

### Allocating budget and resources

Costs for surveillance and response activities, including required equipment, internet connectivity, and labour are supported through the MOPH budget, the health security budget, and local budgets. This domestic funding is enhanced by support from the Global Fund to Fight AIDS, Tuberculosis, and Malaria and additional external support from partners such as the United States President’s Malaria Initiative. The MOPH also works with other agencies to coordinate malaria activities in their relevant domains and identify efficiencies, prioritize tailored activities, and maximize available funding. For example, shared information technology and systems resources across the government support malaria surveillance efforts.

From 2017 to 2020, the MOPH supported the malaria elimination strategy with Thai baht 2.8 billion (USD $88.5 million), which does not include budget spent for health infrastructure and human resources [5]. The Malaria Elimination Steering Committee, comprising members from several ministries and the WHO, develops an integrated budget. Of this budget, 11% is allocated to surveillance activities.

At the local level, PHOs and Local Administrative Organizations (LAOs) in areas with malaria transmission may allocate their own funds to malaria elimination projects. LAOs have subdistrict-level responsibility for public health and welfare issues, including malaria [5, 17]. The 2015 Infectious Disease Act also stipulates that LAOs must conduct malaria surveillance and liaise with district health authorities. LAO committees that prioritize malaria as a community issue are empowered to design projects on disease control and response, based on hyperlocal malaria epidemiology and surveillance data. They also prepare and approve annual budget plans, and they manage funds for emergencies like disease outbreak response or natural disasters.

### Comparisons between the Thailand experience and the China experience

The DVBD adopted the 1-3-7 strategy from China but tailored the strategy’s activities to suit Thailand’s requirements, available resources, and surveillance infrastructure. China included all suspected malaria cases in surveillance activities, which required substantial resources despite the country’s low caseload at rollout in 2012 [19]. In Thailand, because all suspected malaria patients can access blood tests and results within the same day of visit, only confirmed positive cases trigger the 1-3-7 strategy. This not only promotes accurate management of fever patients and efficient use of commodities but also increases the quality of surveillance data by reducing reporting requirements.

Microscopy examination remains the most widely used diagnostic tool for laboratory confirmation of malaria both in Thailand and China. However, with declining incidence, practitioners’ skills in performing and reading blood smears may decrease [9], so RDTs have recently become available in primary health care centers and communities in Thailand. This will allow VHVs to play a greater role in Thailand’s malaria elimination strategy compared with China and also supports Thailand’s system based on confirmed cases rather than suspected cases [20].

Foci classification also varied between the two countries, with four categories in Thailand and three categories in China. In China, areas with at least one confirmed malaria case are classified into three foci categories:

- “Inactive focus” is an area that is unlikely to support transmission because of an absence of vectors;
- “Active focus” is an area with suitable vectors; and
- “Pseudo focus” is a malaria-free area with a case classified as imported [9, 20].

Thailand has been using a foci classification system for several years, throughout both the malaria control and malaria elimination phases, based on malaria source (i.e., indigenous or imported), entomological factors, and environmental characteristics (i.e., unsuitable or suitable for transmission) [7]. Whereas in China both active and pseudo foci prompt RACD, in Thailand RACD screening is carried out only in foci with an index case (A1, A2, or B1).

Recording and reporting requirements were also slightly different because Thailand looked for efficiencies to maximize limited resources. In Thailand, to meet the criteria for foci reporting within 7 days, MIS users may input data through the end of the subsequent week that the case was reported. For example, if the case was identified on Monday of week 1, the VBDU can respond on any day starting on Monday of week 1 through Saturday (i.e., the last day) of week 2. As long as there were no other cases occurring in the same sub-village that week, the MIS will count that case as responded to within 7 days. Because workload for subnational officers can often be a bottleneck to timely surveillance and response, the DVBD applied a workload reducing strategy based on the number of cases in a province. For example, in a high-prevalence province like Tak, which reported 1,685 malaria cases in 2019, only one RACD was required even if more than one index case was reported at the same week in the same sub-village. Because each RACD targets 50 people per index case, coverage was adequate to maintain high-quality surveillance.

### Success factors to Thailand’s 1-3-7 surveillance strategy

The DVBD has heightened the visibility of malaria in Thailand by coordinating a Malaria Steering Committee and working across various government agencies including the Ministry of Interior, Ministry of Foreign Affairs, and Ministry of Defense. To engage policymakers, the team outlined a convincing argument that focused on the benefits of malaria elimination for the development of the country—for example, the DVBD published a cost–benefit analysis that found that every Thai baht invested in malaria elimination can return up to 15 Thai baht to Thailand’s health system, households, and economy [21]. The DVBD also partnered with neighboring countries to share data and policies; this regional approach opened new funding opportunities through the Global Fund Regional Artemisinin-resistance Initiatives. More internally, the DVBD has prioritized malaria surveillance, including the 1-3-7 strategy, in its strategies and budgets, creating an enabling environment from Thailand’s public health leadership. Supporting digital transformation policies have simultaneously catalyzed malaria surveillance infrastructure and success, including developing dashboards that facilitate review of 1-3-7 metrics and catalyze subsequent actions.

At the operational level, Thailand’s malaria officers also have a mobile chat application that is a unique factor in the success of 1-3-7 surveillance. The chat group has over 400 malaria members from across the country and at every level of the health system. The group chat enables continual communication with peers to monitor the malaria situation, exchange experiences, seek recommendations or troubleshooting support, share press releases, and provide fodder for new strategies. Participation of the DVBD and national-level partners also supports more effective monitoring and evaluation. The active members respond to notifications, ask questions and provide answers, and offer motivation and support, making it an efficient platform for monitoring and communication among malaria workers.

A final success factor in Thailand’s implementation is the flexibility of implementing budgets and activities at very local levels. Even though annual budgets are calculated based on the previous year’s activities and unexpected situations often occurred, LAOs were able to work with provincial authorities to revise their local budgets to fit needed activities for the current situation. Because these local authorities know the community situation, this microplanning has been a successful strategy for organizing targeted response even in situations with unanticipated spikes in malaria caseload.

### The future road to elimination

The 1-3-7 strategy has been successfully implemented, and results suggest it has contributed to Thailand’s decline in malaria burden from 14,948 cases in fiscal year 2017 to just 4,421 in fiscal year 2019, representing a 70% reduction in incidence. The DVBD continues to adapt its programme to ensure prompt and high-quality case management, capacity maintenance, and adequate supply of lifesaving commodities. It is also continuing routine data monitoring and wider assessments as needed to verify that the time-bound targets of the 1-3-7 strategy are sustained over time. Senior staff are expanding technical discussions and resources to include prevention of reintroduction. However, the DVBD may also need to consider potential challenges that may emerge as provinces approach very low prevalence.

The MOPH is working toward an integrated system of health services by phasing out vertical VBDUs, VBDCs, and MCs. As staff in these facilities retire, the MOPH is not recruiting replacements so that future patients are directed toward GHS facilities, including health promoting hospitals [13]. The new structure of GHS’s malaria programme is semi-vertical, with the DVBD’s expertise guiding the development of high-quality malaria services across facility types. This guidance includes incorporation of malaria case management into GHS pre-service training, mechanisms of in-service training and professional support through VBDU, and an encouraging career path for GHS trainees [5, 8]. The DVBD will remain involved in the integration process to ensure the high quality of malaria case management and surveillance is maintained through elimination and prevention of reintroduction.

When areas reach very low prevalence, the DVBD may consider supporting provincial authorities to take a stronger role in quality assurance. Additional online training and standard operating procedures could support a more decentralized system to fortify comprehension of protocols despite current challenges caused by the coronavirus disease. An annual online training with certification could be one way to maintain knowledge and skills, share updates, and review surveillance data as fewer health staff interact with suspect malaria cases and have robust malaria case management experience.

As the DVBD’s efforts reduce malaria burden, advocating for necessary funds becomes more challenging. Thailand’s budget for malaria surveillance has changed over time. However, the technical malaria community knows that malaria elimination programmes are more resource-intensive than malaria control, with increasing cost per case. The current global funding landscape is shifting, and external funds available to Thailand are shrinking. This challenge requires the DVBD to be more creative and strategic with identifying sources of financial support. Especially in the described climate of integration, domestic fund-raising is also a challenge because it requires that malaria be prioritized over all other diseases Thailand is working to address.

## Conclusions

In conclusion, the DVBD has successfully tailored the 1-3-7 strategy for malaria surveillance and response to the Thai context with a core objective of accelerating malaria elimination. The time-bound components of this strategy have emphasized the target of zero malaria by 2024, and results from the first few years of implementation show Thailand is on track to reach its goal. The DVBD will continue to track progress and ensure a high quality of care for malaria patients as the programme integrates malaria services into the GHS. This experience may be useful for other countries aiming to eliminate malaria in the region.

## Data Availability

The visualizations supporting the conclusions of this article are available in the Malaria Online repository, http://malaria.ddc.moph.go.th/.

## Abbreviations

API: Annual parasite incidence
BOE: Bureau of Epidemiology
DDC: Department of Disease Control
DVBD: Division of Vector Borne Diseases
GHS: General Health Services programme
GMS: Greater Mekong Subregion
GTS: Global Technical Strategy for Malaria 2016–2030
HH: Household
IRS: Indoor Residual Spraying
ITN: Insecticide-Treated Net
LAO: Local Administrative Organization
MC: Malaria Clinic
MIS: Malaria Information System
MOPH: Ministry of Public Health
MP: Malaria Post
NMES: National Malaria Elimination Strategy
PACD: Passive Case Detection
PCR: Polymerase Chain Reaction
PHO: Provincial Health Office
RACD: Reactive Case Detection
RDT: Rapid Diagnostic Test
SPR: Slide Positive Rate
SRRT: Surveillance and Rapid Response Team
USAID: United States Agency for International Development
USD: U.S. Dollars
VBDC: Vector Borne Disease Center
VBDU: Vector Borne Disease Unit
VHV: Village Health Volunteer
WHO: World Health Organization
